# The value of time-dependent risk predictions in a screening context - a comprehensive simulation analysis validated on German cancer registry data

**DOI:** 10.1186/s12874-022-01718-2

**Published:** 2022-09-10

**Authors:** Vinzenz Voelkel, Teresa Draeger, Sietse van Mossel, Sabine Siesling, Hendrik Koffijberg

**Affiliations:** 1 Tumor Center Regensburg/ University of Regensburg, Institute for Quality Control and Health Services Research, Regensburg, Germany; 2 Department of Health Technology and Services Research, Technical Medical Centre, University of Twente, P.O. Box 217, 7500 AE Enschede, The Netherlands; 3 Department of Research and Development, Netherlands Comprehensive Cancer Organisation (IKNL), Utrecht, The Netherlands

**Keywords:** Personalised screening, Risk prediction, Simulation, Healthcare efficiency, Medical decision making, cancer registries

## Abstract

**Background:**

Risk-prediction tools allow classifying individuals into risk groups based on risk thresholds. Such risk categorization is often used to inform screening schemes by offering screening only to individuals at increased risk of harmful events. Adding information concerning an individual’s risk development over time would allow assessing not just who to screen but also when to screen. This paper illustrates the value of personalised, time-dependent risk predictions to optimize risk-based screening schemes.

**Methods:**

In a simulation analysis, two different time-dependent risk-based screening approaches are compared to another risk-based, but time-independent approach regarding their impact on screening efficiency. For this purpose, 81 scenarios featuring 5000 patients with five consecutive annual risk estimations for a hypothetical disease *D* are simulated, using different parameters to model disease progression and risk distribution. This simulation analysis is validated using a real-world clinical case study based on German breast cancer patients and the INFLUENCE-nomogram for locoregional breast cancer recurrence.

**Results:**

If individual risk estimations were used to personalise screening for a disease D aiming at detecting a 90% of curable cases, more than 20% of screening examinations could be avoided relative to a conventional uninformed approach, depending on the simulated scenario. Whereas an individual but time-independent approach is associated with acceptable saving potentials in case of a relatively homogenous risk distribution, the time-dependent approaches are superior when the complexity of a scenario increases. With slowly progressing diseases, risk-accumulation over time needs to be considered to achieve the highest screening efficiency on population level, for rapidly progressing diseases, an interval-specific approach is superior. The possible benefits of time-dependent risk-based screening were confirmed in the real-world clinical case study.

**Conclusions:**

Appropriate approaches to use time-dependent risk predictions may considerably enhance screening efficiency on individual and population level. Therefore, predicting risk development over time should be supported by future prediction tools and be incorporated in decision algorithms.

**Supplementary Information:**

The online version contains supplementary material available at 10.1186/s12874-022-01718-2.

## Background

With limited financial resources for healthcare, it is essential for society as a whole to reduce unnecessary healthcare spending [1, 2]. The economic benefits of personalised screening have been demonstrated [3, 4]. On individual level, it is important to reduce unnecessary screening examinations which may inflict serious impairments on a patient’s health [5, 6]. In the current striving towards personalised medicine, risk-prediction tools are gaining importance [7]. Adjuvant! Online from the field of breast cancer care and the Framingham risk score for cardiovascular risk are two well-known prediction tools meant to assist clinicians in individualizing and optimizing patient care [7, 8]. Both were built to estimate 10-year risks for their outcome of interest. Like many prediction tools, they concentrate on long prediction intervals since a larger number of events accumulated over time facilitates the fitting of accurate prediction models, and increases their stability [9]. In addition, differentiation between individual risk profiles seems easier when the absolute differences between the corresponding risk estimations are large. For example, a difference of 10% vs. 15% concerning a 10-year risk is likely to be regarded as substantial, whereas a 1.0% vs. 1.5% difference in the annual risks might be deemed less relevant. However, by concentrating on cumulative estimations for the long-term risk, one disregards the longitudinal risk-evolution, which often is a nonlinear function of time [10, 11]. Making use of available information concerning an individual’s expected risk development over time might contribute to improved decision processes. Certain tools, such as CanRisk, and INFLUENCE, can already provide such information [12, 13]. In a screening context, such information can be used to target examinations or interventions not only at high-risk individuals but also at high-risk periods in the lives of these individuals. This information is especially helpful when the severity of untreated disease is likely to increase over time and early detection in a still curable stage is crucial (e.g. screening for malignancies [14–16]). Since guidance on the incorporation of time-dependent risk estimations in the planning of personalised screening schemes is scarce, this paper introduces three promising approaches and evaluates their performance. For this purpose, various scenarios with different disease progression, risk distribution and risk evolution patterns over time are simulated at patient-level. Moreover, all approaches are illustrated in a clinical case study based on the INFLUENCE-nomogram which uses a variety of patient-, tumour -, and treatment characteristics to estimate five consecutive annual risks of locoregional breast cancer recurrence (LRR) in the first 5 years after primary surgery [13]. It was applied to a German breast cancer cohort to explore the yield of the different screening approaches in a real-world context. The analyses presented in this paper are meant to reveal the importance of personalised, time-dependent risk predictions in further optimizing risk-based screening schemes.

## Methods

First, the general definitions underlying the simulation analysis shall be explained. Thereafter, the three simulation parameters that are varied to generate different scenarios are introduced. It follows an overview of the suggested three approaches to design risk-based (and time-dependent) screening schemes, which are compared in each of these scenarios. Lastly, these three approaches are explored in a real-world clinical case study.

### General definitions

Let *D* be a disease that occurs at time *t*_*occ*_ in individuals of an initially disease-free population (*N* = 5000). *D* usually does not cause symptoms in an early, curable stage but can be detected by a hypothetical examination *E* with, which is performed at time *t*_*i*_. The chance that *D* is fully curable decreases with the growing length of the time interval between its occurrence and the following screening examination/detection. In principle, *E* could be performed at any time *t*_*i*_, but for simplicity and to ensure congruency with the real-world clinical case study described later, it may only be done at five fixed time points after the initialization of screening, yielding a maximum of five screening examinations per individual: *t*_*i*_ = *i, i* ∈ {1, 2, 3, 4, 5 years}.

It shall be assumed further that there exists a prediction algorithm *PA* which estimates interval-specific risks for the occurrence of *D* in a single individual *k, k* ∈ [1; 5000] conditional on the fact that this individual did not experience the event in the previous interval*. PA* gives five independent risk estimations *p*_*k,i*_*(D)*, *i* ∈ {1, 2, 3, 4, 5}; each of those covers the risk for the occurrence of *D* within a 1-year interval between two potential time points for screening examinations. The accuracy of *PA* is assumed to be perfect on individual and population level. Therefore, it is not necessary to additionally simulate “real” occurrences of *D* at specified times *t*_*occ*_: if a simulated individual is, for example, assigned a risk *p*_*k,1*_*(D)* = 1% for the first year, this is equivalent to 0.01 theoretical disease occurrences in this interval. The sum of every individual’s conditional risks/ occurrences aggregated over all 5 years yields the expected amount of disease occurrences on population level. Since an annual risk prediction does not provide further information concerning the exact onset time of *D*, it is assumed that *D* occurs on average exactly in the middle of an annual interval: *t*_*occ*_ ∈ {0.5, 1.5, 2.5, 3.5, 4.5 years}.

### Simulation parameters

The performance of risk-based screening approaches likely depends on the scenario they are applied to, which is characterised by features of the target population or the disease. To reflect certain key aspects of these features, three parameters are employed to simulate five individual consecutive annual risk predictions for three kinds of disease progression patterns and *N* = 5000 patients.

#### Coefficient of variation of mean risk *(cvmr)* across five years

**Fig. 1:**
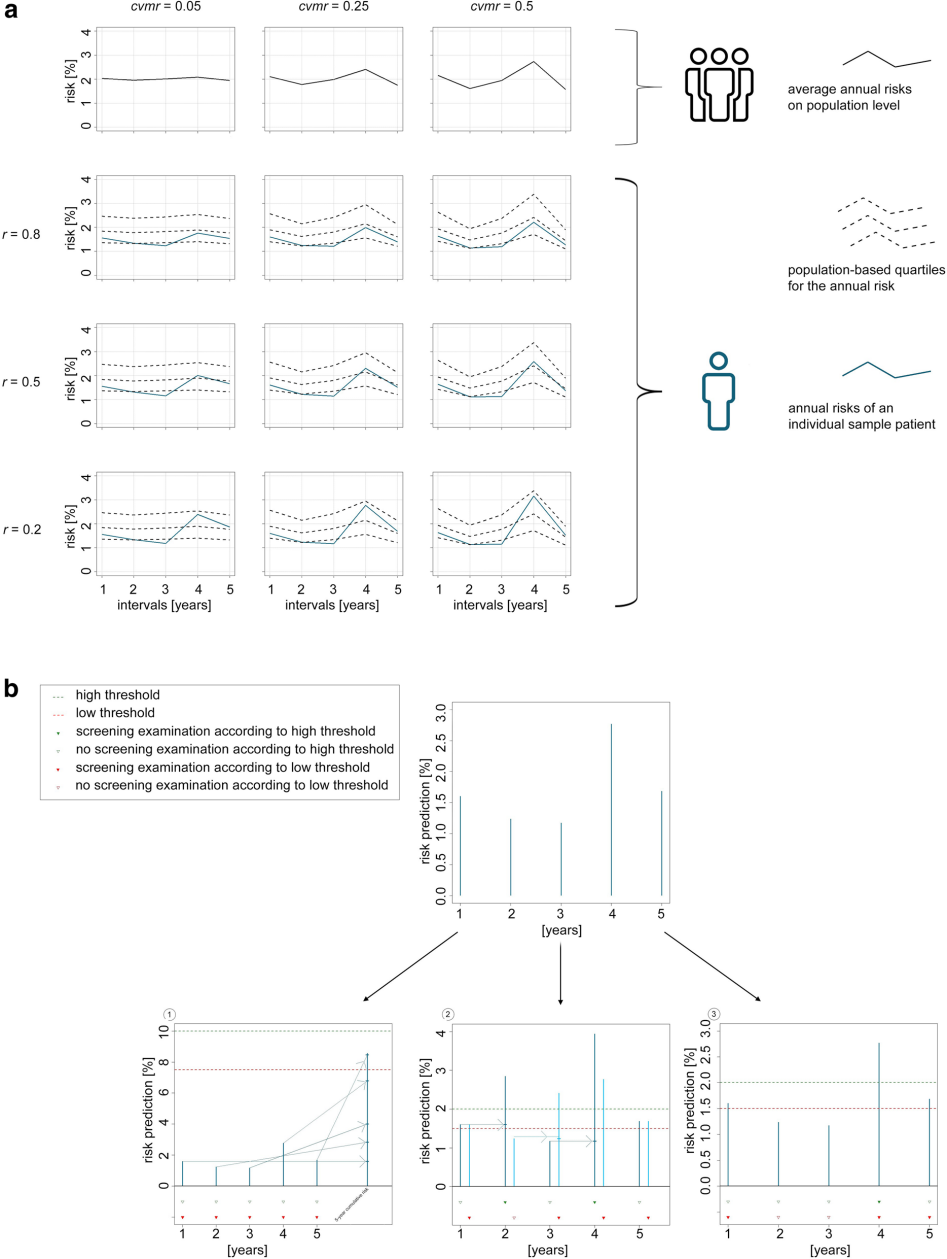
**a** Showcase risk distribution over time, dependent on *r* and *cvmr. Cvmr:* coefficient of variation*; r:* Pearson correlation coefficient. **b** Visualization of decision strategies. (1) cumulative approach / CA: interval-specific risks (dark blue bars) are added up (dashed arrows): if the sum exceeds the chosen threshold, a screening examination is done. (2) approach with interval-specific reevaluation / CAIR: interval-specific risks are added up (dashed arrows) until the chosen threshold is reached, leading to a screening examination (cumulation process according to high threshold: dark blue bars; cumulation process according to low threshold: light blue bars). (3) interval-specific approach / ISA: if an interval-specific risk (dark blue bars) exceeds the threshold, a screening examination is done. *Cvmr:* coefficient of variation*; r:* Pearson correlation coefficient

At population level, the average cumulative 5-year risk per individual is set to 10% since many diseases show comparable incidence rates in clinical practice [17, 18]. If *cvmr* was zero*,* meaning no variation of the mean risk, the mean annual risk would be perfectly stable at 2% in each year. Setting *cvmr* to a value larger than zero induces variation concerning the distribution of the five mean annual risks over time*;* for example, the mean annual risks might be > 2% in years 1 and 2, and < 2% in years 3–5, while the average cumulative risk still sums up to 10% over 5 years. For the different simulation scenarios, *cvmr* was set to 0.5 (equivalent to a standard deviation of the mean annual risk, *sdmr,* of 1%) representing high, to 0.25 (equivalent to a *sdmr* of 0.5%) representing intermediate, and to 0.05 (equivalent to a *sdmr* of 0.1%) representing low variation of the mean annual risks over time (Fig. 1a, top line). Having generated the mean annual risks like this, the individual risk estimations per year were assumed to be normally distributed around the population average risk for the same year. The corresponding standard deviations were assumed to be directly inversely correlated to the log-value for a year’s mean risk on population level.

#### Parameter 2: Pearson correlation coefficient *r*

A patient with a relatively high value for *p*_*k,1*_*(D)* in the first year likely features relatively high values *p*_*k,i*_*(D)*, i ∈ [2, 5] for the subsequent years as well since their risk profile always relies on the same fixed predictors. However, the influence of one or more predictors may change over time causing shifts in the relative size of consecutive risk estimations. In this simulation, the correlation parameter *r* was set to 0.2 to simulate low, to 0.5 to simulate intermediate, and to 0.8 to simulate high correlation between the five annual risk predictions on individual level. To maintain simplicity, it was assumed that *r* is constant between all five annual predictions. Figure 1a, lines two to four, illustrates the influence of *r* on the simulated annual risks of a randomly chosen sample patient, given the underlying population-level risk distribution, which is dependent on *cvmr.* Since *r* also influences whether changes over time in the relative risk of individuals, compared to other individuals, are common (low correlation) or rare (high correlation), the population-based quartiles for the annual risks are displayed as a reference.

#### Parameter 3: disease progression *dp*

Screening aims at early detection of disease manifestations to avoid severe or permanent damage, possibly even leading to premature death. While time to detection is always a crucial factor in this context, progression rates to an irreversible state differ between diseases. Sometimes there is still a fair chance to cure a disease several years after its first occurrence, sometimes the time window closes considerably faster, after 1 year or even earlier. In this simulation, it was assumed that progression to an irreversible disease state follows an exponential function returning an individual’s chance to be fully cured if her or his disease *D,* which occurred at time *t*_*occ*_*,* is detected at time *t*_*i*_: $p\left({D}_{curable}\right)={dp}^{\left({t}_i-{t}_{occ}\right)}$. The function’s basis *dp* was set to 0.6 representing slow, to 0.3 representing intermediate, and to 0.1 representing fast disease progression.

In total, we defined 27 scenarios in which screening approaches will be compared. These represent all possible value-combinations of the three parameters *cvmr*, *r*, and *dp.*

### Screening approaches

Performing a screening examination at time *t*_*i*_ is assumed to result in the detection of an individual’s accumulated theoretical occurrences of *D.* It is supposed to detect each new occurrence of the disease since the last screening examination or the beginning of the screening programme if it concerns the first screening examination. If the maximum of five screening examinations was performed in all individuals, every occurrence of D, *p*_*k,i*_*(D),* would be detected after half a year on average. In this study’s setup, such a full screening approach would, by definition, yield the highest possible number of disease detections in a still curable state (*n*_*max*_(*D*_*curable*_)) on individual and population level.

Due to economic or psychological constraints, a full screening approach might not be desired or feasible. However, reducing the number of screening examinations on individual and population level inevitably leads to delayed detection of *D* in some individuals, which is associated with a lower chance of curability. If a screening examination is supposed to detect not only occurrences of *D* from the previous, but also earlier years, the actual number of diseases which are detected in a still curable state (*n*_*actual*_(*D*_*curable*_)) decreases for earlier occurrences, dependent on the parameter *dp* and the actual detection delay *t*_*i*_ *- t*_*occ*_*.*

Therefore, before different risk-based screening approaches are proposed and compared, it is important to define the desired target detection rate *(tdr)*, which is the number of detected occurrences of *D* in a curable state on population level, divided by the maximum number of disease detections $: tdr=\frac{n_{actual}\left({D}_{curable}\right)}{n_{max}\left({D}_{curable}\right)}$ . All screening approaches shall be compared regarding the number of screening examinations which is necessary to achieve exactly the predefined value for *tdr.* Since in the view of ethical considerations it seems unlikely that clinicians would accept an overly high “missing rate”, it was decided to assess three settings with a *tdr* of 80*,* 90, and 95%. Based on this, the following three approaches to develop individualised screening schemes are proposed and will be compared:

#### Cumulative approach (CA

All five annual risk predictions of *PA* for a patient are summed. A risk threshold corresponding to the pre-defined target detection rate *tdr* is determined and applied to the cumulative risk to discern between high-risk and low-risk patients. High-risk patients are assigned a screening examination after each year, while screening is completely omitted in low-risk patients. (Fig. 1b-1, lower-left panel).

#### Cumulative approach with interval-wise reevaluation (CAIR)

**Fig. 2:**
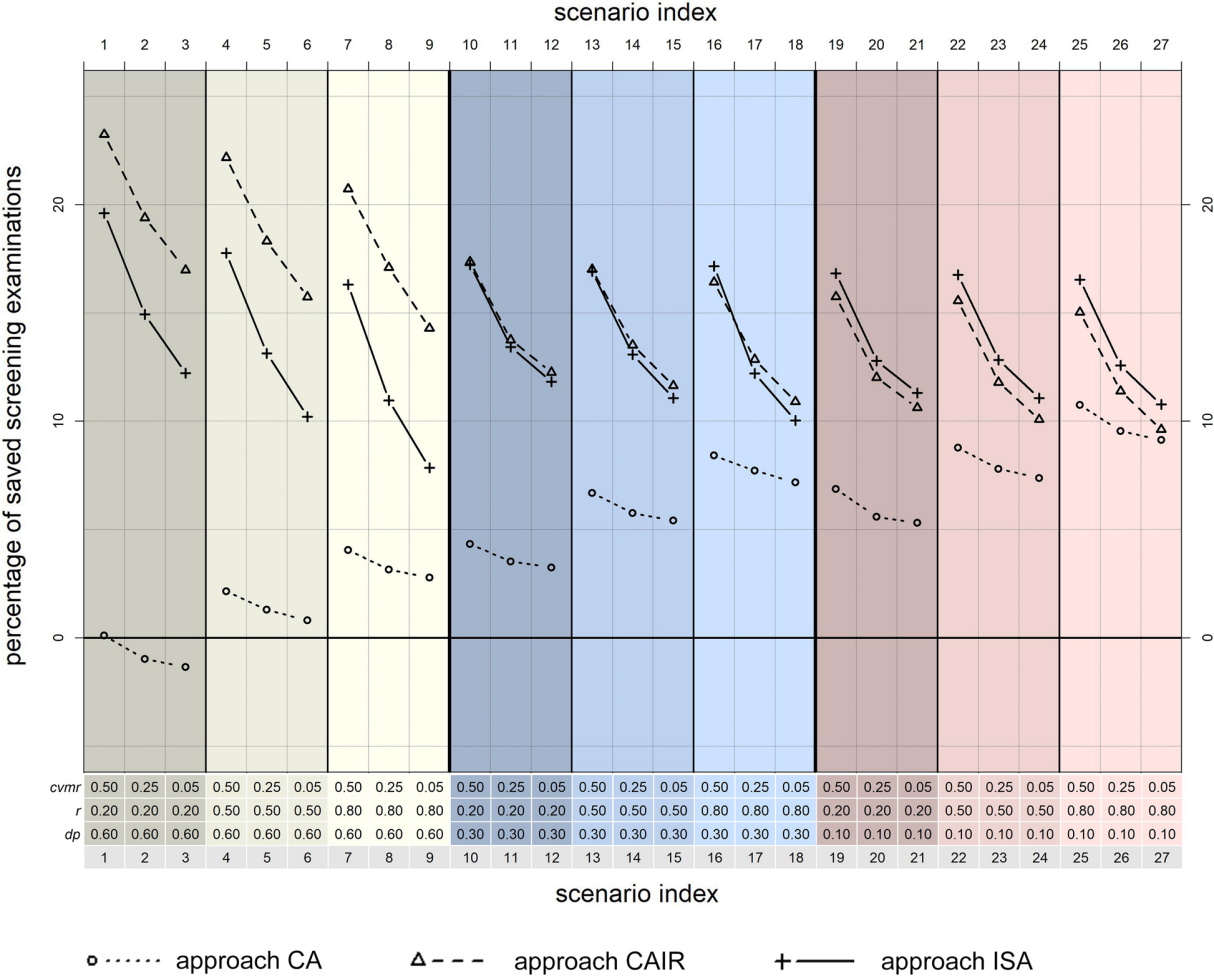
Comparing the screening-efficiency of the proposed screening approaches (*tdr* of 90%, for all 27 scenarios). CA: cumulative approach; CAIR: cumulative approach with interval-specific reevaluation; ISA: interval-specific approach. *dp:* disease progression; *cvmr:* coefficient of variation of mean risk*; r:* Pearson correlation coefficient; *tdr:* target detection rate

A patient’s annual risk predictions are summed up until a threshold corresponding to the pre-defined target detection rate *tdr* is reached. Consequently, this patient is assigned a screening examination which will be performed at the end of the last year contained in the sum. If a screening examination was performed, the cumulation process starts anew, beginning with the risk of the following year and continuing until the threshold is reached again. (Fig. 1b-2, lower-middle panel).

#### Interval-specific approach (ISA)

**Fig. 3:**
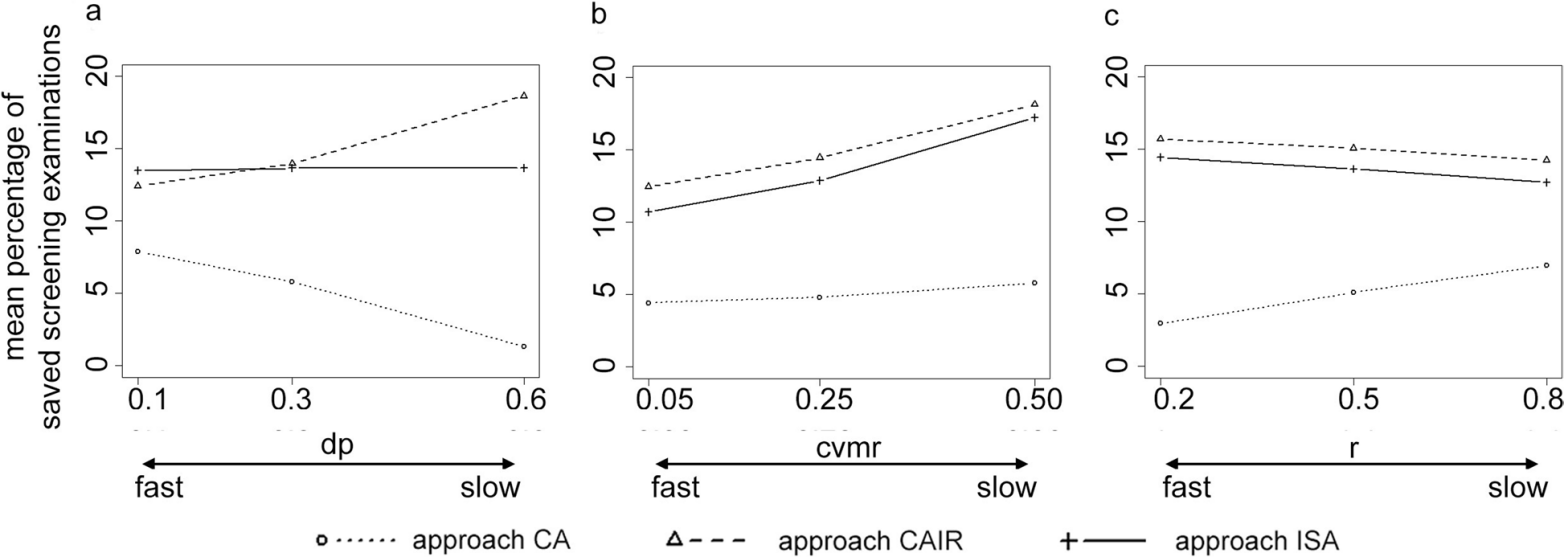
Influence of *dp, cvmr,* and *r* on the proposed screening approaches’ efficiency (*tdr* of 90%). **a** The influence of *dp,* averaged over all simulated values for *cvmr* and *r.* **b** The influence of *cvmr,* averaged over all simulated values for *dp* and *r.* **c** The influence of *r*, averaged over all simulated values for *cvmr* and *dp.* CA: cumulative approach; CAIR: cumulative approach with interval-specific reevaluation; ISA: interval-specific approach. *dp:* disease progression; *cvmr:* coefficient of variation of mean risk*; r:* Pearson correlation coefficient; *tdr:* target detection rate

A patient is assigned a screening examination after every year in which her or his annual risk exceeds a threshold resulting in the pre-defined target detection rate *tdr.* (Fig. 1b-3, lower-right panel).

To compare these three approaches, a reference is required. While it seems unrealistic that anyone would rely on chance to allocate screening examinations, a random approach (RA) is the most appropriate reference for comparing approaches that are less extensive than full screening. The previously proposed three approaches will only provide added value in daily clinical practice if they require fewer screening examinations to achieve the predefined target detection rate *tdr* compared to screening allocation in the most uninformed, random way. The percentage of potentially avoided inefficient screening examinations of the approaches CA, CAIR, and ISA relative to the reference approach RA is calculated for all 27 simulated scenarios and three target detection rates. To ensure stability of the results and reduce stochastic uncertainty, each scenario was evaluated 100 times and results were averaged.

### Clinical case study

Currently, there do not exist many prediction tools which facilitate the calculation of time-dependent risks. One of them is the INFLUENCE-nomogram [13]. Based on a patient’s age, tumour size, nodal involvement, grade, estrogen−/ progesterone-status, multifocality, radiotherapy, chemotherapy, and endocrine therapy, it estimates conditional annual risks of developing a locoregional breast cancer recurrence (defined as reappearance of the tumour in the ipsilateral breast, chest wall or regional lymph nodes) within 5 years after diagnosis. The INFLUENCE-nomogram’s algorithm is based on data of more than 37,000 Dutch patients diagnosed with early breast cancer between 2003 and 2006. Its external validity was recently demonstrated by applying it on a cohort of 6520 breast cancer patients diagnosed between 2000 and 2012 obtained from Tumorzentrum Regensburg (Institute for Quality Control and Health Services Research of University of Regensburg), a clinical cancer registry in Germany [19]. The same cohort is used in this study to examine how follow-up patterns and rates of missed locoregional recurrence (LRR) events might look like, if the approaches CA, CAIR, and ISA were applied in clinical practice.

Following actual guideline recommendations, the potential time points for screening/ follow-up examinations were set to 1, 2, 3, 4, and 5 years, like in the previously described simulation analyses. While the parameters *cvmr* and *r* are fixed given the real individual risk predictions of INFLUENCE, an assumption concerning disease progression of locoregional breast cancer recurrences had to be made. Based on clinical evidence that early detection yields significant advantages in survival [14], it was assumed to be at least intermediate and the parameter *dp* was again set to 0.3. Additionally, the target detection rate *tdr* was set to 90%. In contrast to the simulation analyses, the assumption of perfectly accurate predictions is not valid in a real-world example. Moreover, there do not exist theoretical partial disease occurrences in a single individual. Instead, the real recurrence events observed in the German population were counted as detected by the consecutive follow-up examination which would have been assigned to a patient. Of course, there does not exist a perfect examination procedure in breast cancer follow-up. Since the sensitivity of the usually employed – and sometimes combined - diagnostic procedures varies between 65% for mammography, around 90% for ultrasound and up to 100% for MRI, an overall examination sensitivity of 80% was incorporated in the analyses [20]. To quantify the impact of such a screening examination, the real detection delays were determined and used to calculate a patient’s chance to be fully cured.

For all analyses, *R* version 3.5.1 (R Foundation for Statistical Computing, Vienna, Austria; http://www.R-project.org/) was used.

## Results

### Simulation analysis

In total, 3 × 27 scenarios featuring 5000 patients with five annual risks/theoretical occurrences of D at an average individual five-year risk/ occurrence rate of 10% were simulated. This represents all possible value combinations of the parameters disease progression (*dp* ∈ {0.1, 0.3, 0.6}), coefficient of variation of mean risk (*cvmr* ∈ {0.05, 0.25, 0.5}), and Pearson correlation coefficient (*r*: ∈ {0.2, 0.5, 0.8}) at three different target detection rates (*tdr* ∈ {80, 90, 95%}). In the following paragraphs, the performances of the three proposed screening approaches CA (cumulative approach), CAIR (cumulative approach with interval-specific reevaluation), and ISA (interval-specific approach) relative to RA (random approach) are analyzed in detail, using 27 scenarios with a fixed *tdr* of 90%. The corresponding results for *tdr* = 80% and *tdr* = 95% are briefly mentioned at the end of this section and can be found in the supplementary material.

Using approach RA to achieve *tdr* = 90% requires on average 41,678 screening examinations at *dp* = 0.6, 43,670 at *dp* = 0.3, and 44,613 at *dp* = 0.1. These values serve as reference for all percentages presented in the following comparisons. Across all scenarios, the risk-based, but time-independent approach CA is associated with the smallest potential to save screening examinations. It ranges between − 1.3% in scenario 3 (*dp* = 0.6, *cvmr* = 0.05, *r* = 0.2) and 10.7% in scenario 25 (*dp* = 0.1, *cvmr* = 0.5, *r* = 0.8). Moreover, CA is always inferior to the other two proposed approaches CAIR and ISA. Especially when disease progression is slow (*dp* = 0.6, cf. scenarios 1–9), only limited or even negative gains in screening efficiency can be observed. (Fig. 2).

The two risk-based, time dependent approaches CAIR and ISA are always associated with a considerable, positive saving potential of at least 7.8%, regardless of the parameters *cvmr* and *r.* Approach CAIR yields its maximum saving potential in scenario 1 (23.2%, *dp* = 0.6, *cvmr* = 0.5, *r* = 0.2), and its minimum in scenario 27 (9.6%, *dp* = 0.1, *cvmr* = 0.05, *r* = 0.8). The saving potential of approach ISA ranges between 19.6%, also in scenario 1, and 7.8% in scenario 9 (*dp* = 0.6, *cvmr* = 0.05, *r* = 0.8). For high values of *dp* (0.6, equivalent to slow disease progression, cf. scenarios 1–9), CAIR’s performance is always superior to approach ISA. For intermediate disease progression (*dp* = 0.3, cf. scenarios 10–18), both approaches are equally efficient, while for high disease progression (*dp* = 0.1, cf. scenarios 19–27), approach ISA is always slightly superior. (Fig. 2).

Overall, the saving potential varies considerably dependent on the features of the underlying disease and population, represented by the simulation parameters *dp*, *cvmr*, and *r*. Notably, the influence of these parameters is quite heterogenous for the three proposed approaches. Figure 3a shows the percentage of potential savings per approach, averaged over all scenarios with the same parameter *dp.* The slower the disease progression (the higher *dp*), the higher is the saving potential of approach CAIR: it increases from an average value of 12.4 to 18.7%. The opposite is true for approach CA. Its saving potential decreases at the same time from 7.9 to 1.3%. The saving potential of approach ISA is not sensitive to changes in *dp* and remains almost constant at an average value of 13.6%.

The risk-variation over time represented by *cvmr* mainly affects the performance of the time-dependent approaches CAIR and ISA, whereas the approach CA is not very sensitive to changes in this parameter. If all scenarios with the same *cvmr* are averaged, the increase from *cvmr* = 0.05 to *cvmr* = 0.5 is associated with an additional saving potential of 5.6% for approach CAIR, 6.5% for approach ISA, and only 1.4% for approach CA (Fig. 3b). In contrast, the correlation *r* between the annual risk estimations mainly influences the performance of the time-independent approach CA. When averaging across all scenarios with the same value *r*, the number of potential savings increases from 3.0% at *r* = 0.2 to 7.0% at *r = 0.8*. Approach CAIR and approach ISA are less affected by the parameter *r* (Fig. 3c).

Looking at all 27 scenarios separately again, one can see that the differences between the three proposed approaches concerning potentially avoided inefficient screening examinations decrease with faster disease progression (smaller *dp*), smaller risk variation over time (smaller *cvmr*), and higher correlation between the individual annual risks (higher *r*). The corresponding analyses with *tdr* = 80% and *tdr* = 95% yield quite similar results, while the absolute saving potentials are considerably higher, or lower, respectively. The exact results of these analyses can be found in the supplementary figures [see Additional files 1 and 2].

### Clinical case study

**Fig. 4:**
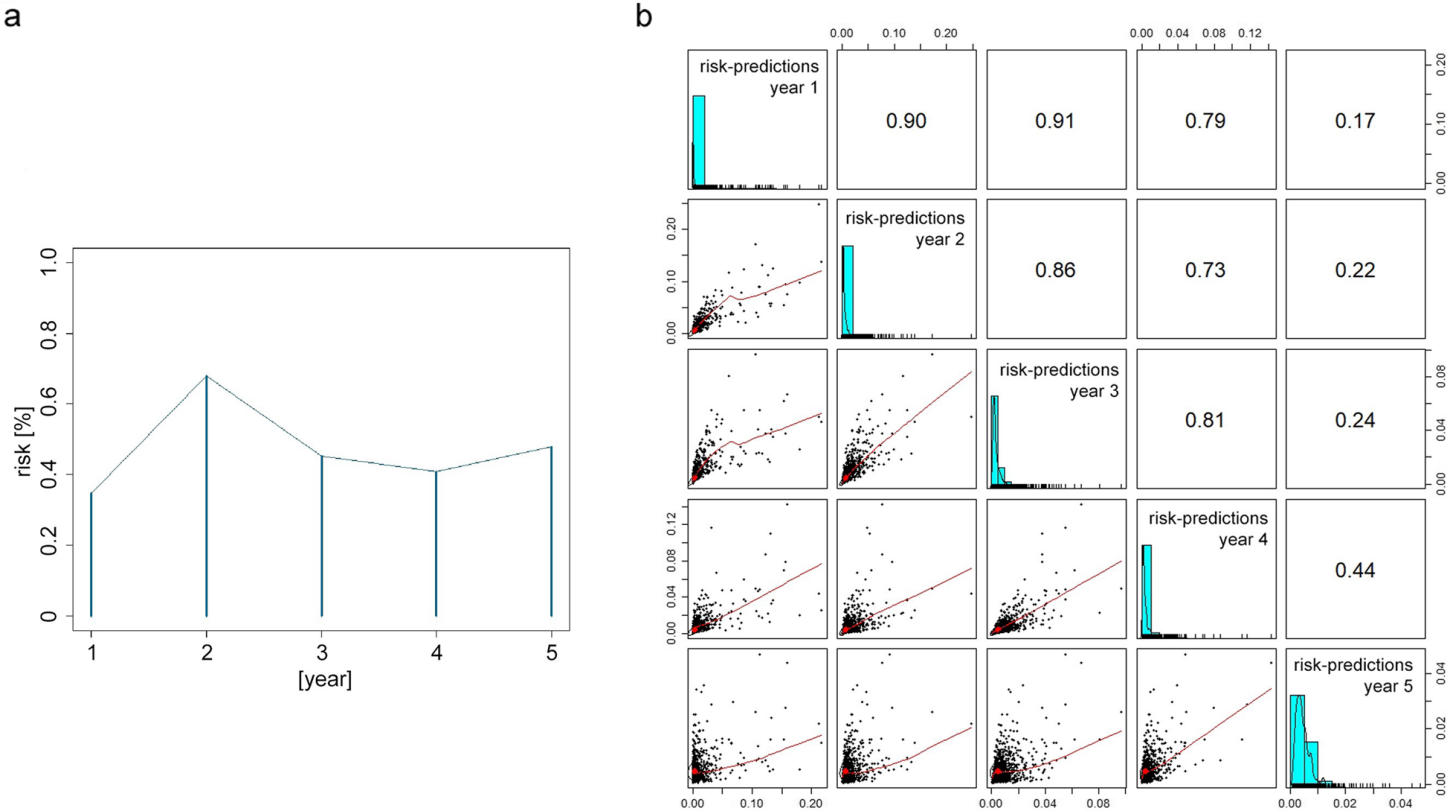
The INFLUENCE-nomogram’s predictions for a German patient-cohort. **a** Mean predicted annual risk over time. **b** Correlation between interval-specific (annual) risk predictions. The row and column mappings are shown in the matrix diagonal together with histograms indicating the individual annual risk predictions’ distribution. Matrix lower left triangle: scatterplots showing the relation between individual risk predictions for two different years. Matrix upper right triangle: Spearman correlation coefficients between individual risk predictions for two different years

The average LRR-risk for the 6520 German patients estimated by the INFLUENCE nomogram is 2.2% for all 5 years. It reaches its minimum in year 1 (0.35%), and its maximum in year 2 (0.68%, Fig. 4a), with a mean annual value of 0.47% (*cvmr* = 0.24). Taking all individual risk predictions into account, the mean Spearman correlation coefficient between the annual risk estimations was $\overline{r}$ = 0.61. According to the correlation matrix, correlation is very high between year 1 and the years 2 and 3, high between year 1 and year 4, and low between year 1 and year 5. In general, correlation declined with increasing time periods between risk estimates (Fig. 4b).

If a disease progression rate of *dp* = 0.3 is assumed and the maximum of 5 follow-up examinations per patient (as recommended by the current guideline [21]) would be performed, one could expect to detect 92 out of 184 observed recurrence events in a still fully curable state. If a *tdr* of 90% is aspired this number decreases slightly to an expected detection rate of 82 still curable recurrence events. To achieve this aim, on average 28,392 follow-ups would have been necessary if the approach RA was chosen. Applying approaches CA, CAIR, and ISA would yield a potential saving of 4542 (16.0%), 4857 (17.1%) and 6465 (22.8%) follow-ups, compared to approach RA (Table 1).

**Table 1:** Comparison of the proposed screening approaches using German patients’ INFLUENCE-predictions (*tdr* of 90%)

strategy	n (follow-up)	% inefficient examinations avoided	n (detected)
RA	28,392	Ref.	82
CA	23,850	16.0	
CAIR	23,535	17.1	
ISA	21,927	22.8	

*RA* Reference approach, *CA* Cumulative approach, *CAIR* Cumulative approach with interval-specific reevaluation, *ISA* Interval-specific approach

**Fig. 5:**
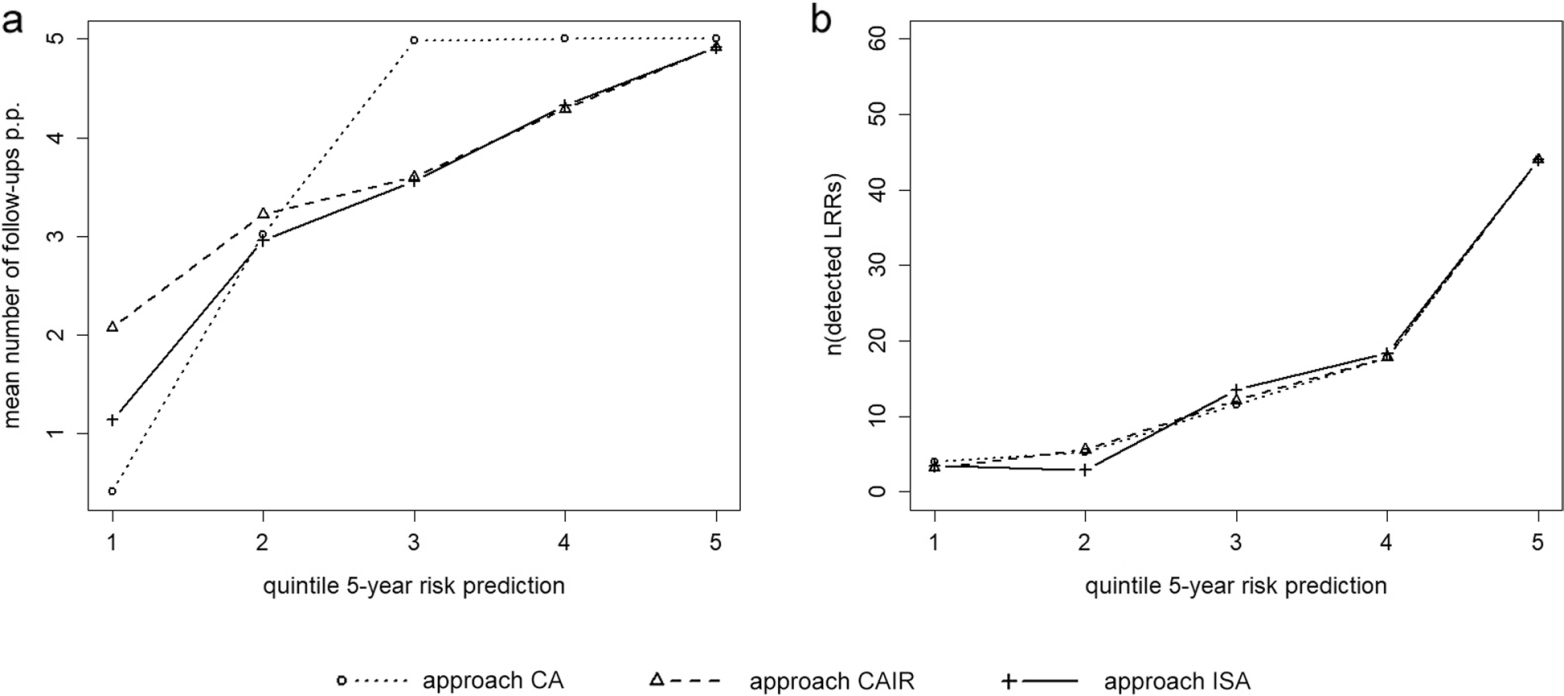
The impact of the proposed screening approaches on patient-level. **a** Mean number of follow-ups per patient (p.p.) stratified for 5-year risk quintiles. **b** Number of detected LRRs per 5-year risk quintile. 5-year risk quintiles: 1 [0.14%; 0.66%], 2 [0.66%; 0.96%], 3 [0.96%; 1.56%], 4 [1.56%; 3.03%], 5 [3.03%; 38.80%]. *CA*: cumulative approach; *CAIR*: cumulative approach with interval-specific re-evaluation; *ISA*: interval-specific approach

On an individual level, a patient who belongs to the lowest risk quintile regarding her 5-year overall risk estimation (between 0.14 and 0.66%) would on average receive 0.4 follow-up examinations over all 5 years in approach CA, 2.1 examinations in approach CAIR, and 1.1 examinations in approach ISA. Patients belonging to the third quintile with a 5-year risk estimation between 0.96 and 1.56% could expect to receive 5 follow-up examinations in approach CA, and 3.6 in approach CAIR and ISA. Patients of the highest quintile with a 5-year risk estimation between 3.03 and 38.80% were likely to receive 5 follow-up examinations according to approach CA, and 4.9 according to the approaches CAIR and ISA (Fig. 5a). Regarding the actual detection rates, more than 50% of the detected disease occurrences would be diagnosed in patients belonging to the highest risk quintile, regardless of the applied approach. Concerning the patients from other risk quintiles, minor differences in the expected detections rates exist between the three approaches, but no clear trends can be observed (Fig. 5b).

## Discussion

This study evaluates the potential of time-dependent risk-predictions to enhance screening efficiency for an abstract disease D by comparing different approaches on how to use them. For this purpose, 3 × 27 scenarios with different values for disease progression (*dp*), risk distribution (*cvmr*), and risk correlation (*r*) at three different target detection rates (*tdr*) are simulated. Given a *tdr* of 90% and dependent on different values for the other simulation parameters, the simple risk-based but time-independent cumulative approach CA could save up to 10.7% of screening examinations compared to the uninformed random approach RA. The two approaches CAIR and ISA outperform CA in all simulated scenarios by using information concerning the risk development over time; their maximal saving potential within this study’s setup is 23.2 and 19.6%, respectively. In scenarios with slow disease progression, CAIR is superior to ISA, in scenarios with fast disease progression ISA has a slightly higher potential to improve screening efficiency.

There are many reasons to personalise patient care, especially in a screening context. Every unnecessary treatment or examination is a potential threat to a patient’s quality of life [22, 23]. In the worst case, overtreatment due to false-positive results can even cause permanent physical or psychological damage [24, 25]. Moreover, it is obvious that with limited – financial – healthcare resources it is essential for society as a whole to reduce unnecessary expenses [1, 2]. During recent years, many studies showed the economic benefits of personalised screening [3, 4], but without taking into account that an individual’s risk for a disease or certain adverse event may change over time. While several studies revealed that risk-based dynamic monitoring in the presence of new examination results during screening is a promising new approach to further reduce overtreatment [26–28], the present study focused on the varying influence of fixed risk predictors.

This paper was designed to provide the reader with a versatile insight into the potential of time-dependent risk predictions in a screening context and does not intend to cover all aspects of a comprehensive health economic analysis such as treatment costs or the burden of missed detections. Since the focus of this study is on the most efficient usage of time-dependent risk predictions in general, it does not account for detailed features of a specific disease or diagnostic procedure. The assumption that disease progression strictly follows an exponential function acknowledges that time to detection usually is crucial. However, this is a simplification of reality and does not account for the fact that often inter-individual variation concerning disease progression exists, which might influence screening benefits [29]. Such simplifications regarding disease progression are often needed, since only tracking disease progression after detection without intervening when proven effective treatment options are available is unethical.

To further maintain simplicity, only the two most important outcome measures of a screening setting were incorporated: the detection rate for a hypothetical disease *D*, represented by the target detection rate *tdr,* and the corresponding number of necessary screening examinations as a surrogate parameter for efficiency. Moreover, in reality, there obviously exists no prediction algorithm with a perfect accuracy of 100% and screening tests also do not have perfect sensitivity and specificity. These are clear limitations of this study, which may lead to an overestimation of the efficiency gain that may be realized when using time-dependent risk predictions to tailor screening moments. Including uncertainty in prediction and test outcomes in our simulation may have avoided these limitations, and moved simulation results closer to real-world case studies, but this is beyond the scope of this study and should be topic of further research. The restriction to five annual risk predictions and five corresponding time-points, at which screening examinations are feasible, is another tribute to simplicity. Thus, the results could directly be transferred to the presented real-world clinical case study and were easier to interpret, even if one is not familiar with the topic of time-dependent risk predictions. However, by changing the number and length of consecutive prediction intervals, the findings can be projected to all kinds of primary, secondary and tertiary screening situations. Furthermore, the presented approach may be valuable not only when optimizing screening for recurrences over short time periods but also for optimizing screening for first occurrences of a disease over long time periods, if time-dependent risks of disease occurrence can be predicted.

The results of this study show that individual, time-dependent risk predictions are only the first step towards personalised care. The specific approach to use them has a considerable impact on the expected benefit. Simple discrimination between high- and low-risk patients (approach CA) according to cumulative risk estimations is always likely to be a suboptimal solution when information about the risk development over time is available. It might cause dangerous underdiagnosis and -treatment in patients just below and ineffective overtreatment in patients just above the chosen threshold. Especially if a disease with a slow progression rate shall be detected, at least one screening examination at the end of a large interval could make a huge difference, while full screening is not associated with substantial benefits. In such situations, even screening allocation at random might be more efficient.

The faster the disease progresses, the more important is early detection. Approach ISA exclusively uses the most recent risk prediction as a decision criterion in favour or against screening examinations. Thus, it accounts for the fact that chances to cure a disease that occurred in a certain interval (e.g., a year) which is already covered by a previous risk prediction, are very limited anyway. CAIR is a “hybrid” approach. It uses time-dependent risk predictions and aggregates them if the chosen threshold is not reached in a single year/ interval. This makes CAIR the most efficient approach for slowly progressing diseases with larger detection windows.

It has been shown that the advantage of personalised, time-dependent screening approaches over the plain cumulative approach CA increases when risk variation over time is higher and the correlation between an individual’s annual/ interval-specific risk predictions is lower. This coincides with what intuition might tell us: the more heterogenous a screening situation is, the more can be gained from tailoring follow-up schemes to single individuals and specific periods in which they are at high risk. In “simple” situations with relatively uniform risk predictions, one must weigh the expected gain in screening efficiency yielded by time-dependent approaches against the additional efforts it takes to implement them.

The presented real-world clinical case study on breast cancer follow-up is not subject to some of the simplifying assumptions made in the simulation analysis, like the perfect accuracy of the used prediction algorithm or the employed screening examinations. Nevertheless, it supports the external validity of the simulation’s results. All three proposed approaches yield superior results regarding screening efficiency compared to approach RA, demonstrating the added value of personalised and time-dependent risk predictions. The rank-order of the three compared approaches changed, though, relative to the most comparable scenario 14 from the simulation analyses (characteristics of scenario 14 vs. characteristics of real world clinical case study: cvmr: 0.24 vs. 0.25, r: 0.61 vs. 0.5, dp: 0.3 vs. 0.3. In other words, in the real-world case study, the variation in mean risk over time (cvmr) was similar, the correlation between within individual risks over time (r) was slightly lower, and the speed of disease progression (dp) was similar, as in hypothetical scenario 14.). While the incorporation of a real-world examination sensitivity might play a role in this context, the overall performance of INFLUENCE also needs to be considered. Voelkel et al. showed that the prediction tools’ sensitivity and accuracy is acceptable if applied to the German breast cancer patients, but could be improved [19].

Personalised screening approaches aim to enhance screening efficiency on population level by optimizing screening allocation and, thus, avoiding unnecessary examinations with the potential to cause physical and psychological harm. Such tailoring will result in certain individuals being classified as “low risk”, and therefore receiving no or very limited screening examinations. This is exactly what can be observed in the real-world clinical case study. The average number of an individual’s follow-up examinations varies considerably between the observed risk quintiles, dependent on the chosen screening approach. Remarkably, the three approaches do not differ equally substantially concerning their risk-dependent detection rates, which documents the efficacy of the proposed personalised approaches not only on population-, but also on individual level. While it is obvious that high-risk patients could benefit from personalised screening approaches, the detection rates in low-risk patients of the real-world clinical case study do not decline substantially when omitting screening examinations in this group. However, a detailed assessment of the concrete clinical scenario, including ethical and social judgments next to economic considerations and more accurate assumptions concerning disease progression is indispensable before a specific screening approach is adapted. Furthermore, decision-makers always need to consider the patients’ individual preferences and their presumed compliance before implementing new screening schemes in daily clinical practice.

## Conclusions

Personalised, time-dependent risk predictions corresponding to a disease’s detection window can considerably enhance screening efficiency by tailoring screening examinations to high-risk periods. However, choosing the optimal approach to use such predictions is essential, and requires a careful evaluation of the concrete screening context. Since risk development over time is ignored by most of the existing prediction algorithms, further research efforts towards this direction are recommended.

## Supplementary Information

**Additional file 1. **Comparing the screening-efficiency of the proposed screening approaches (*tdr* of 80%, for all 27 scenarios). CA: cumulative approach; CAIR: cumulative approach with interval-specific reevaluation; ISA: interval-specific approach. *dp:* disease progression; *cvmr:* coefficient of variation of mean risk*; r:* Pearson correlation coefficient; *tdr:* target detection rate.

**Additional file 2. **Comparing the screening-efficiency of the proposed screening approaches (*tdr* of 95%, for all 27 scenarios). CA: cumulative approach; CAIR: cumulative approach with interval-specific reevaluation; ISA: interval-specific approach. *dp:* disease progression; *cvmr:* coefficient of variation of mean risk*; r:* Pearson correlation coefficient; *tdr:* target detection rate.

## Data Availability

The simulation outcomes generated in the current study are available from the corresponding author on reasonable request. Readers interested in the code of the simulation analysis may contact the corresponding author. The German cancer registry data used for analyses in this study are not owned or controlled by the study authors and cannot be made publicly available due to legal reasons. These data can be requested from the German cancer registry.
