# Supplementary figures and images for: The value of time-dependent risk predictions in a screening context - a comprehensive simulation analysis validated on German cancer registry data
Source: BMC Med Res Methodol. 2022 Sep 10;22:239. doi: 10.1186/s12874-022-01718-2 (PMC9464381; doi:10.1186/s12874-022-01718-2)

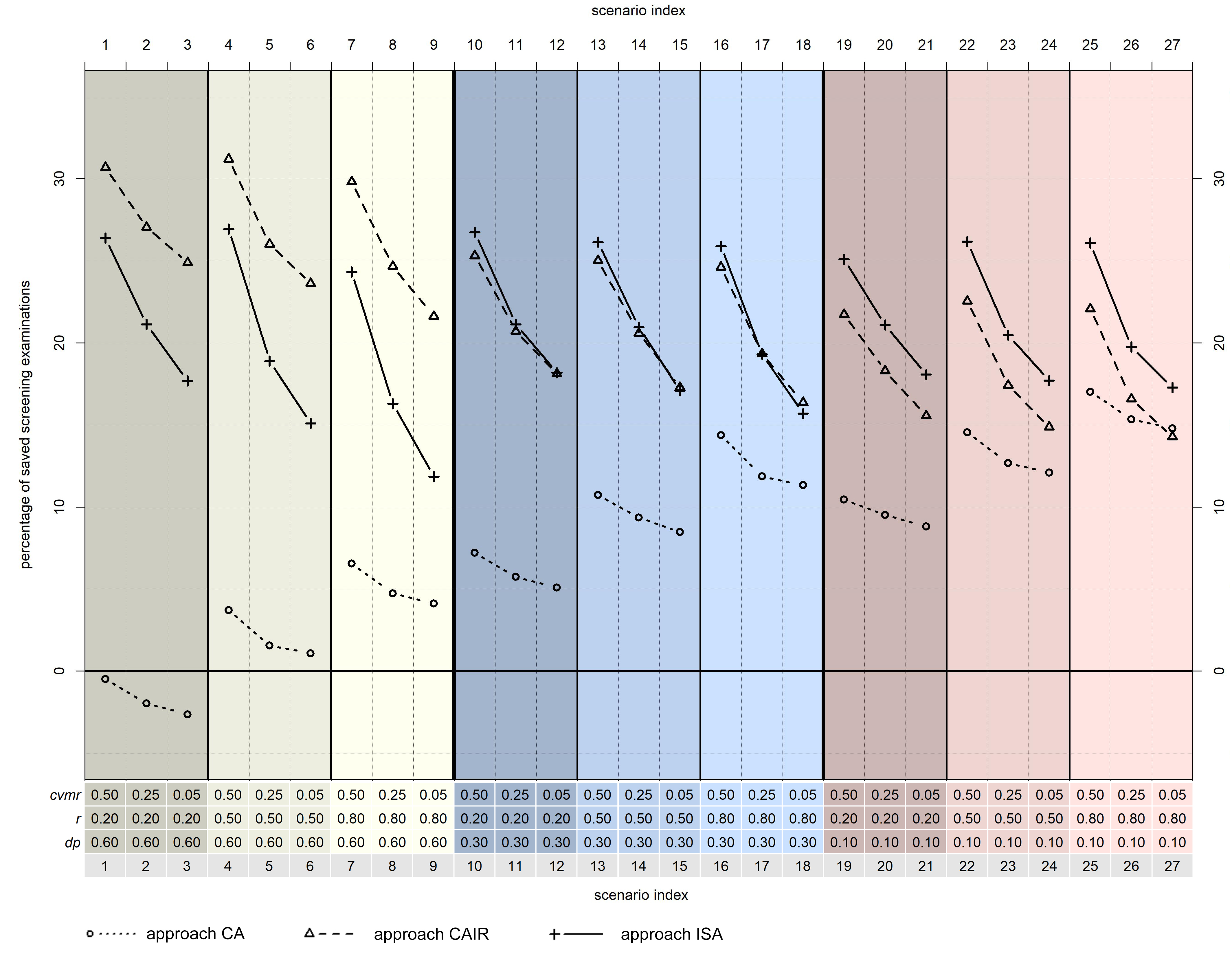

Supplement: Supplementary file 1 — Additional file 1. Comparing the screening-efficiency of the proposed screening approaches (tdr of 80%, for all 27 scenarios). CA: cumulative approach; CAIR: cumulative approach with interval-specific reevaluation; ISA: interval-specific approach. dp: disease progression; cvmr: coefficient of variation of mean risk; r: Pearson correlation coefficient; tdr: target detection rate. [file 12874_2022_1718_MOESM1_ESM.jpg]

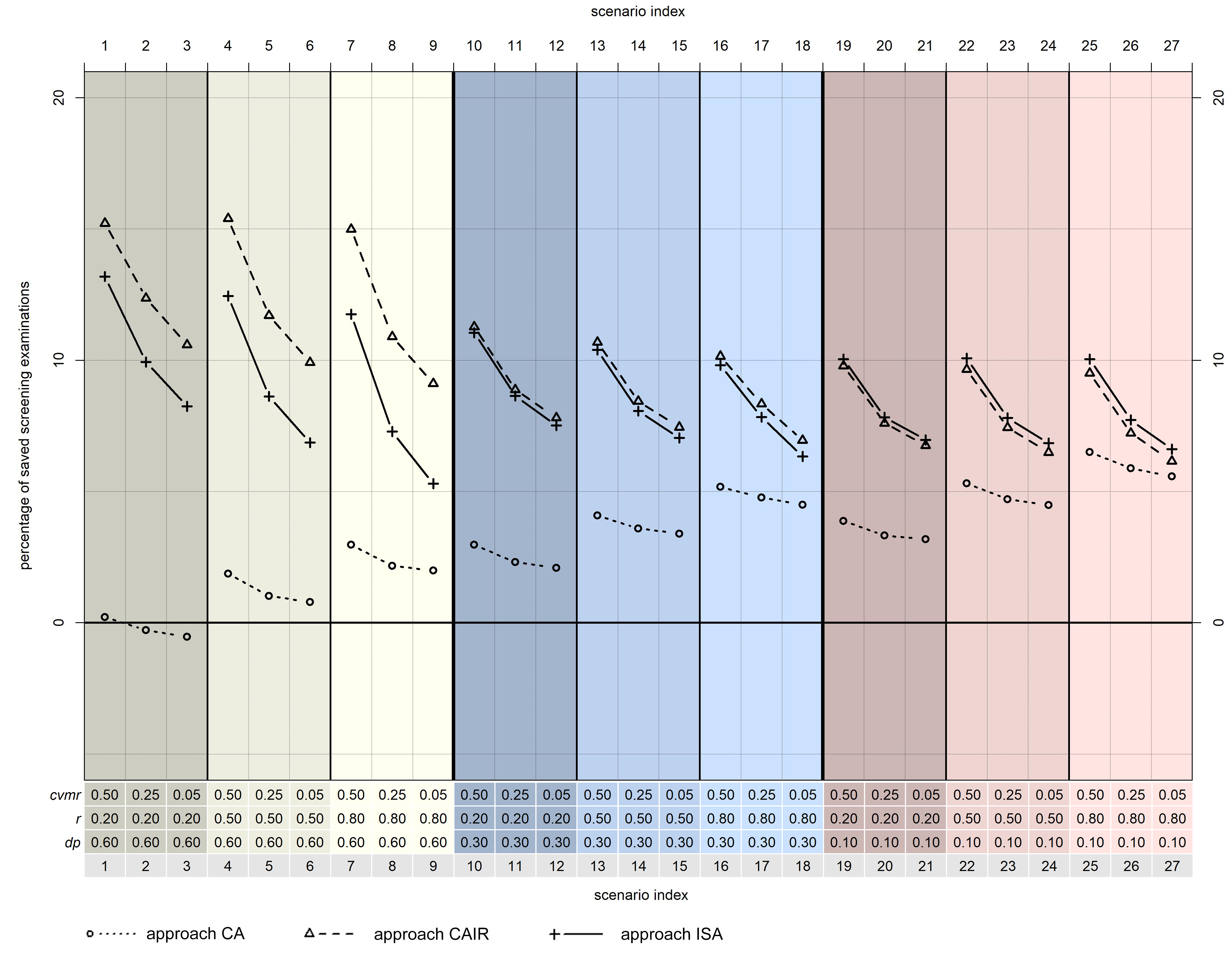

Supplement: Supplementary file 2 — Additional file 2. Comparing the screening-efficiency of the proposed screening approaches (tdr of 95%, for all 27 scenarios). CA: cumulative approach; CAIR: cumulative approach with interval-specific reevaluation; ISA: interval-specific approach. dp: disease progression; cvmr: coefficient of variation of mean risk; r: Pearson correlation coefficient; tdr: target detection rate. [file 12874_2022_1718_MOESM2_ESM.jpg]
